# Supplementary material for: Functional dissection of the ARGONAUTE7 promoter
Source: Plant Direct. 2019 Jan 2;3(1):e00102. doi: 10.1002/pld3.102 (PMC6508778; doi:10.1002/pld3.102)
Supplement: Supplementary file 1 [file PLD3-3-e00102-s001.pdf]

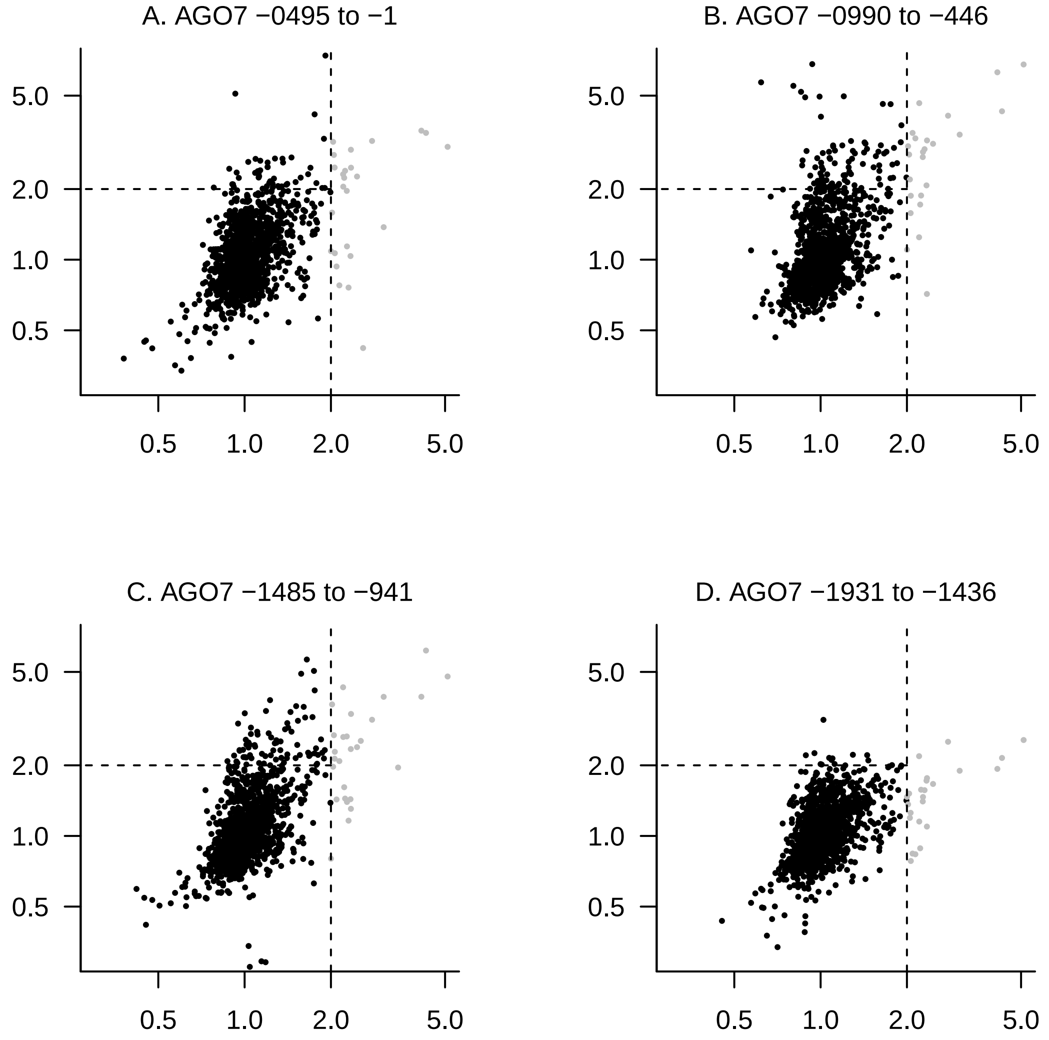

Figure S1. Scatterplots of β-gal activities with likely nonspecific activatiors indicated for *AGO7* promoter fragment screens. Panel B is equivalent to Figure 1C.

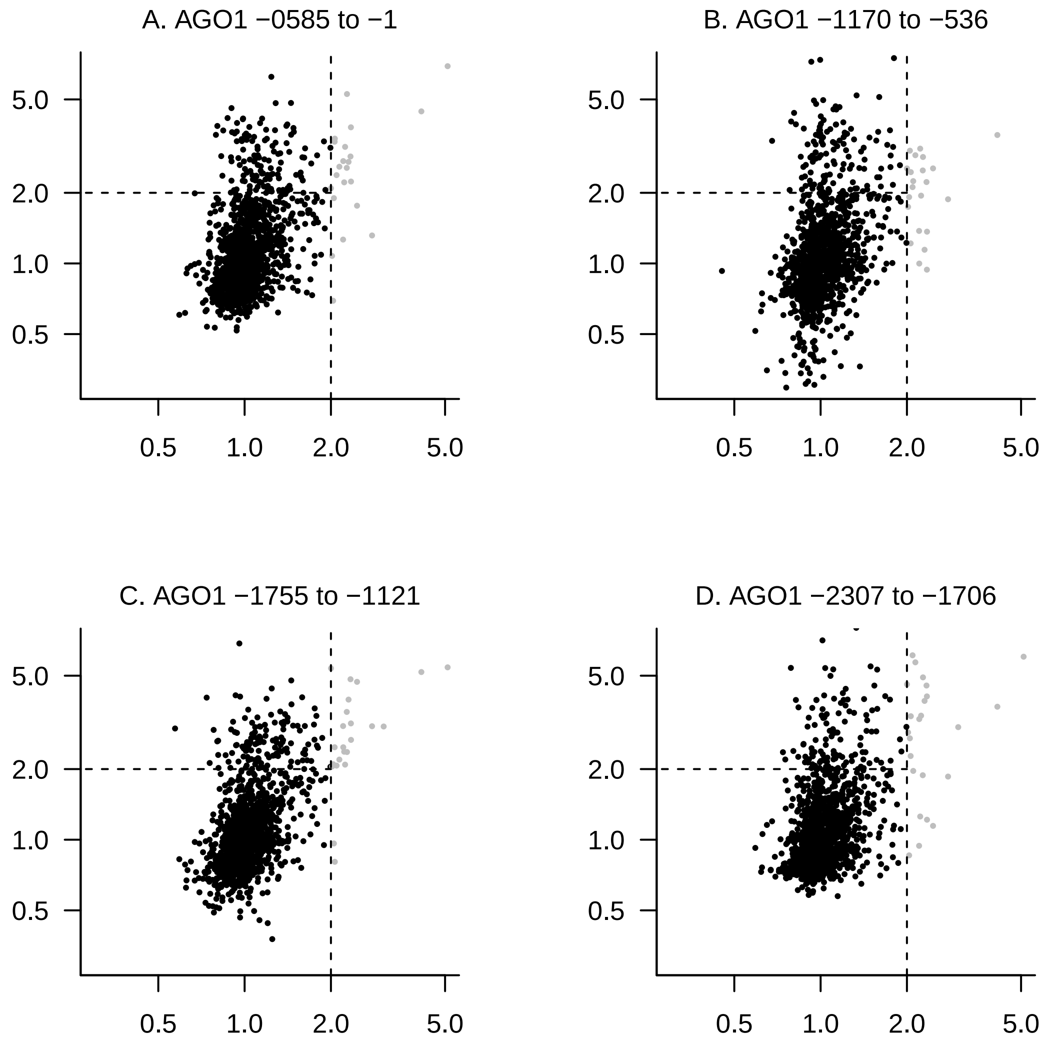

Figure S2. Scatterplots of β-gal activities with likely nonspecific activatiors indicated as in Figure 1C for *AGO1* promoter fragment screens.

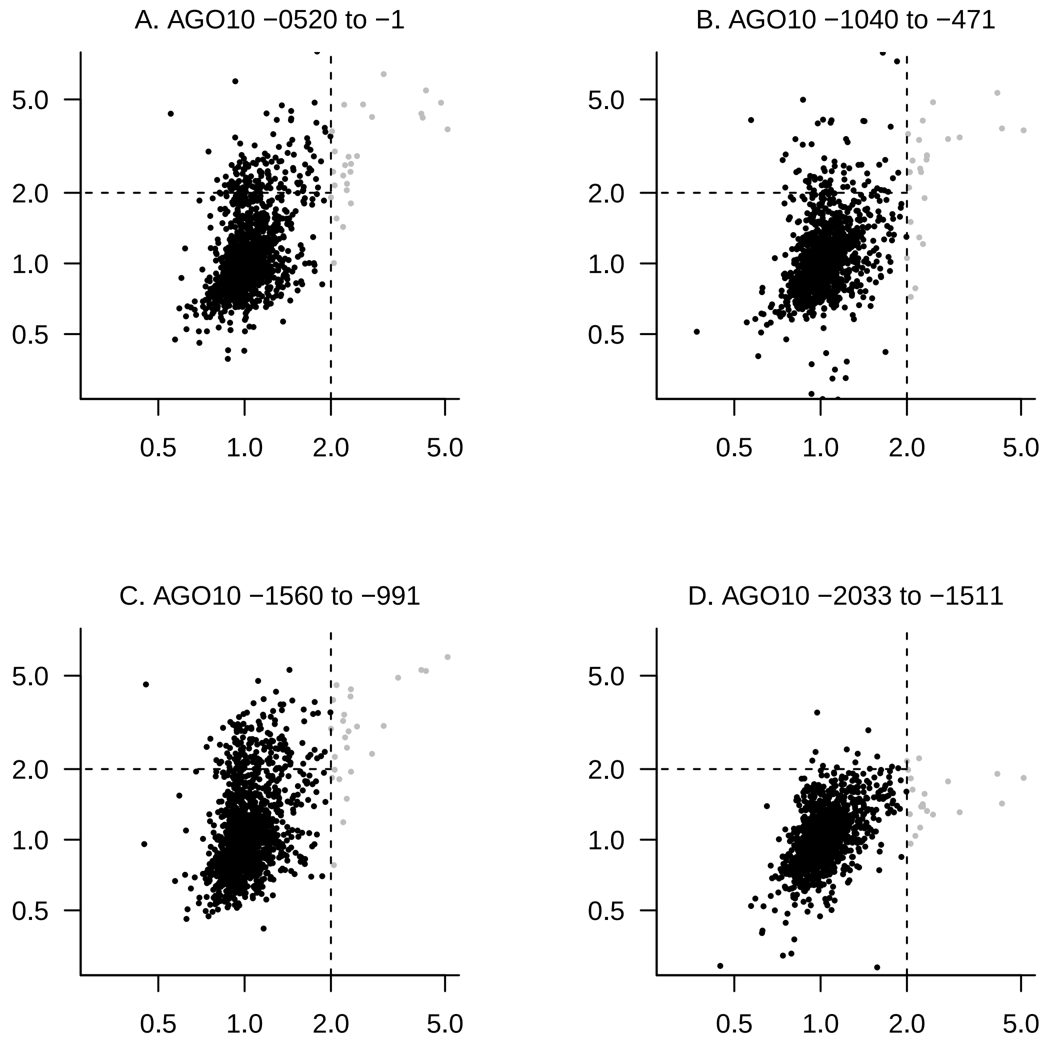

Figure S3. Scatterplots of β-gal activities with likely nonspecific activatiors indicated as in Figure 1C for *AGO10* promoter fragment screens.

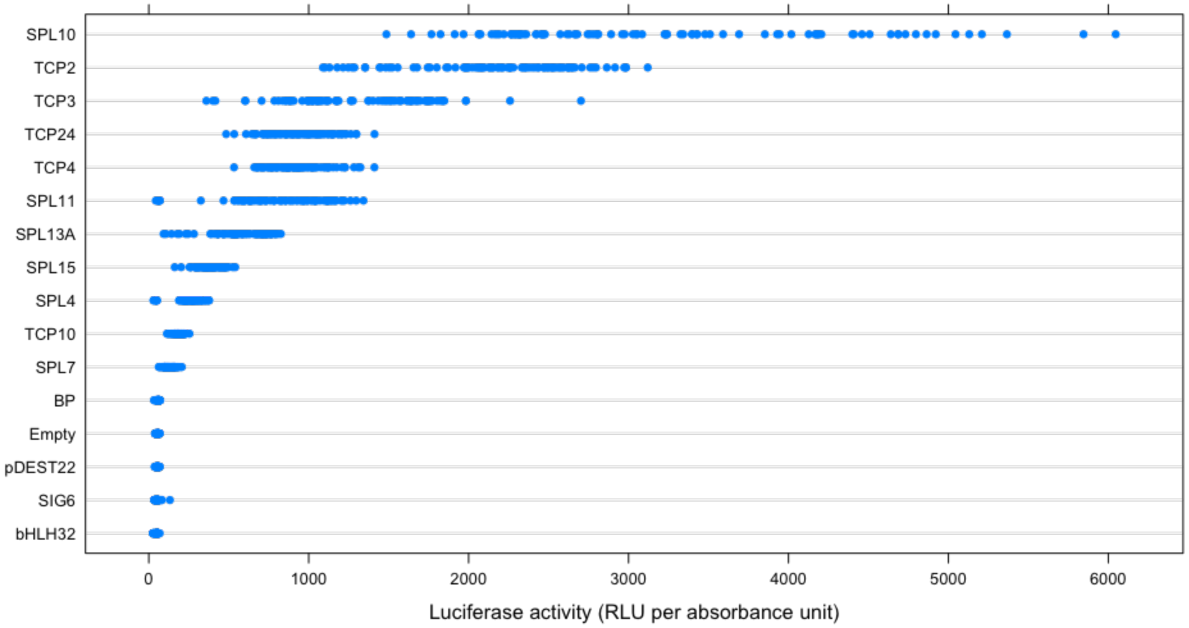

Figure S4. Targeted Y1H assay using *Gaussia* luciferase reporter, quantified in terms of relative luminescence units per absorbance unit at 600 nm. TFs were tested against the *AGO7* -990/-446 region and are displayed in order by mean reporter activity.

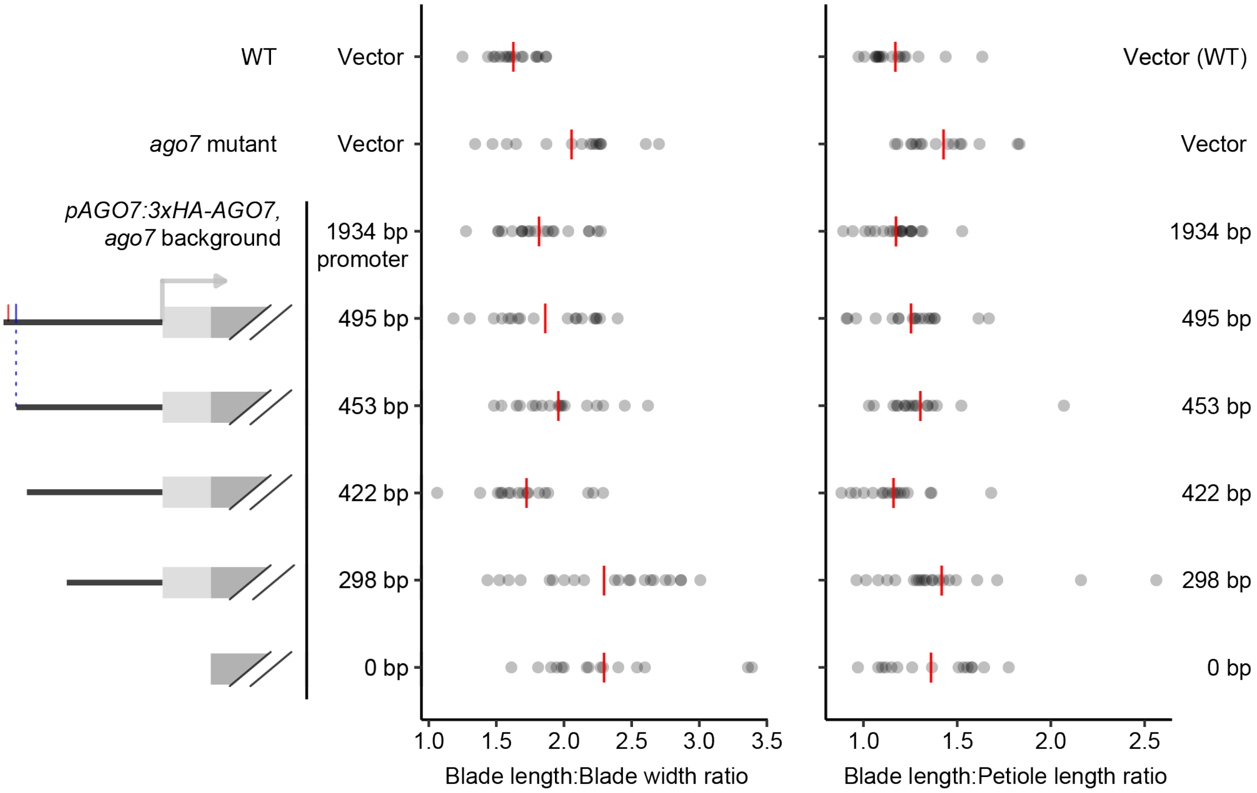

Figure S5. Complementation of *ago7* leaf shape defects, quantified based on leaf blade length-to-width ratio (left) and leaf-blade-length to petiole-length ratio (right) measured for true leaf 6 with calipers on days 28 to 30 days post-stratification. Each datapoint shows the ratio for a distinct primary transformant. Red lines indicate the mean for each genotype.

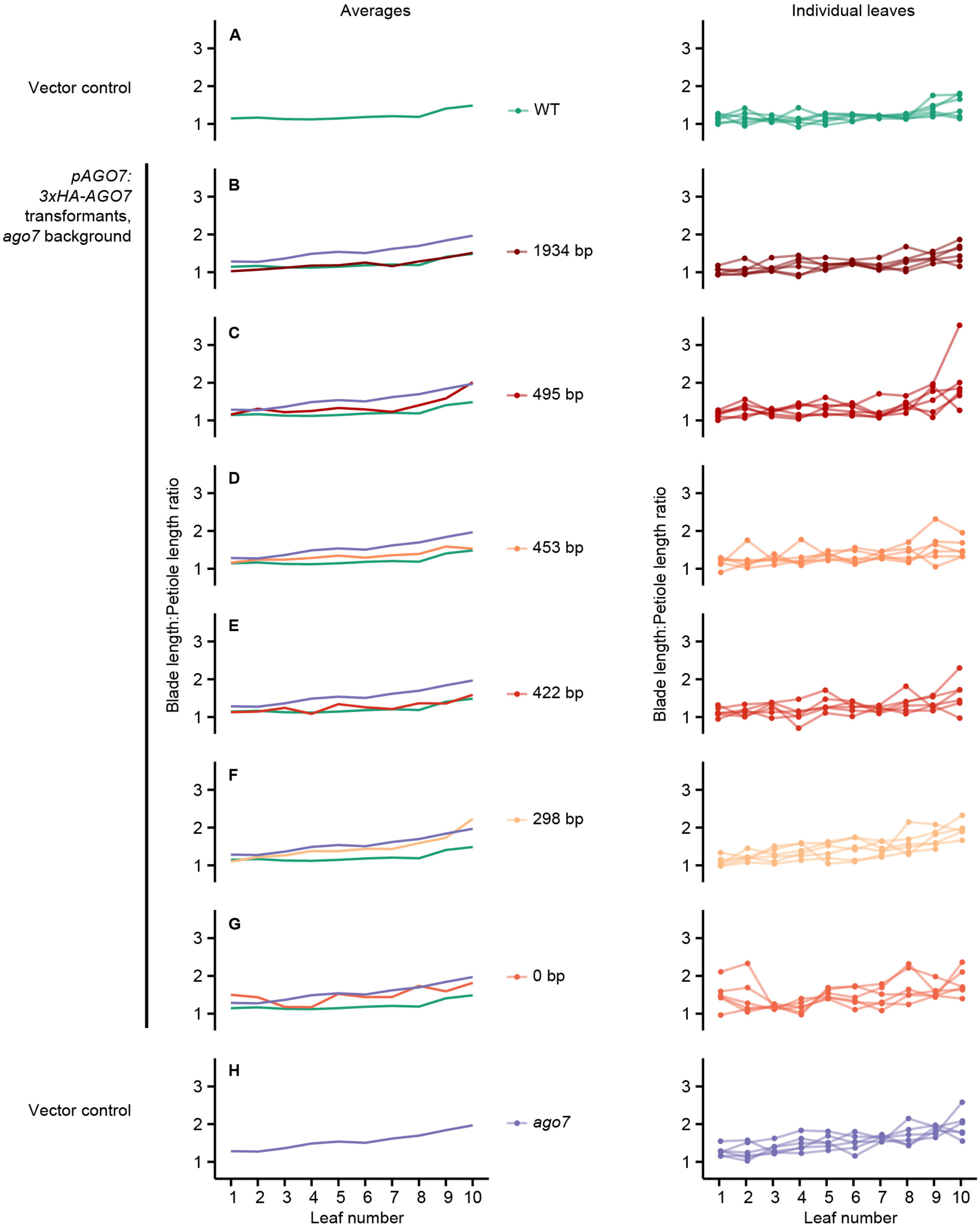

Figure S6. Complementation of *ago7* leaf shape defects, quantified based on leaf blade length to petiole length ratio. Panel layout is as in **??**.

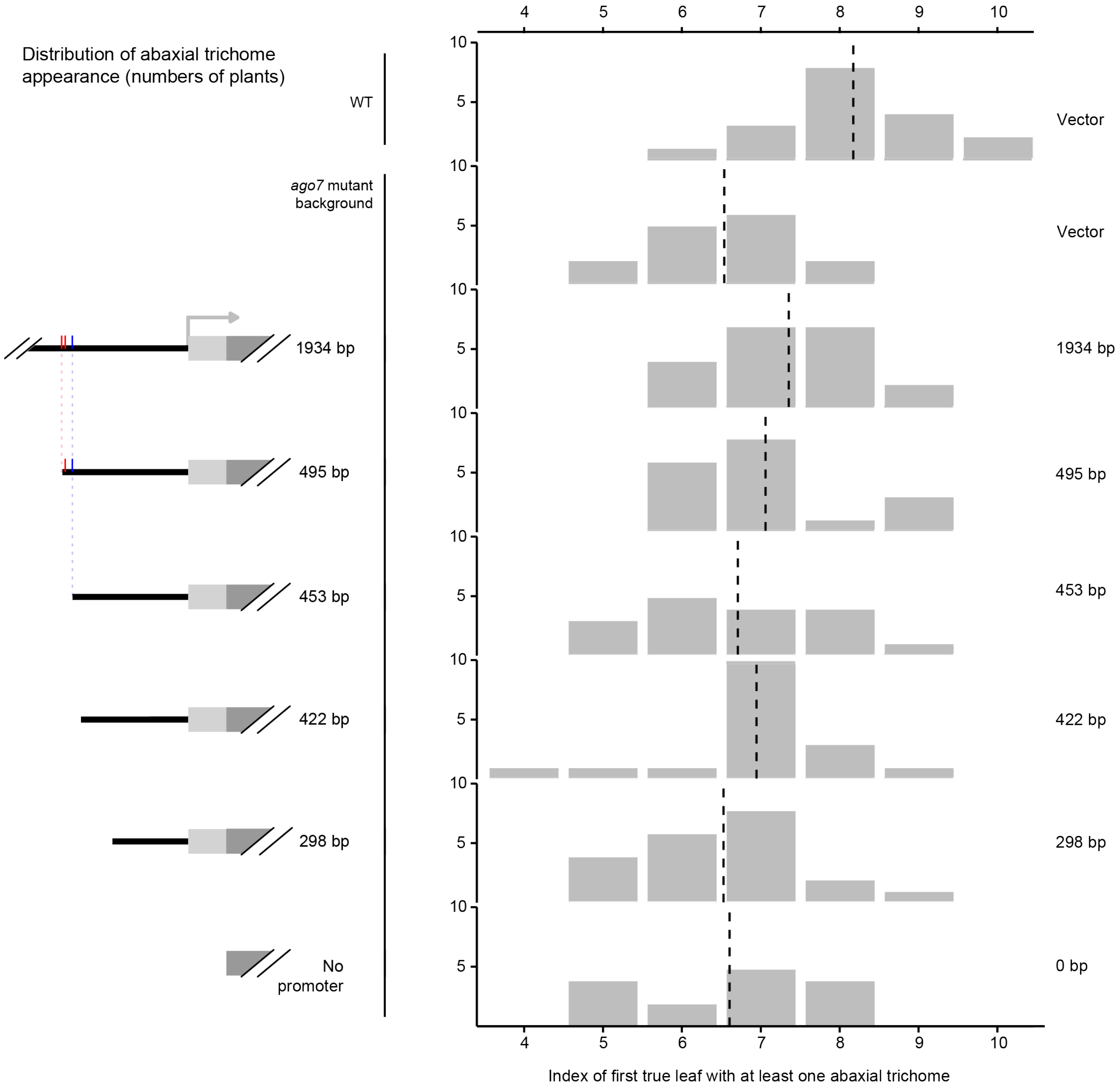

Figure S7. Assay for complementation of *ago7* early abaxial trichome appearance phenotype with 3xHA-AGO7 transgenes driven by truncated versions of the *AGO7* promoter. Dashed lines indicate the mean for each genotype. Core SPL (red) and TCP (blue) binding sites are indicated with tick marks.
