# Supplementary material for: Functional dissection of the ARGONAUTE7 promoter
Source: Plant Direct. 2019 Jan 2;3(1):e00102. doi: 10.1002/pld3.102 (PMC6508778; doi:10.1002/pld3.102)
Supplement: Supplementary file 2 [file PLD3-3-e00102-s002.pdf]

Reviewer #1:

This manuscript describes a yeast 1-hybrid experiment to identify Arabidopsis transcription factors that bind to the AGO1, AGO7, and AGO10 promoters. Most of the work focuses on AGO7. Several transcription factors are identified binding to the AGO7 promoter, including members of the SPL and TCP gene families. These interactions were confirmed with reporter assays in yeast. Promoter-bashing experiments in Arabidopsis, however, showed largely negative results in terms of the effect of these SPL and TCP binding sites on AGO7 transcriptional pattern and complementation.

This is very clearly written manuscript. The experimental designs are sound and the conclusions drawn are appropriate given the data shown. The figures are very clear. Overall this is sound and original work and to this reviewer it meets the criteria for publication in Plant Direct.

I have a few small comments for the authors to consider:

1. Figure 1b: TCP2, TCP4, and TCP10 are hits. On Figure 3, TCP10 is not shown. Is this because there was no empirically identified PWM available for TCP10? Please add an explanation about this to the text.

> Correct: to our knowledge no empirically determined PWM for TCP10 (AT2G31070) is publicly available. A note has been added to the text.

2. Can Figure 4 be re-plotted with the same format as Figure 2? I found figure 2's format, with the control and experimental data shown on the same plot, to be easier to understand.

> Figure 4 has been re-plotted to match Figure 2.

3. Figure 5: The contrast on these images was quite low on my device. Can this be improved?

> We have revised the figure: we enhanced contrast for all photomicrographs equally using ImageJ.

Also, I had to re-read the figure legend several times to understand why there were two columns of images for each construct. I suggest you find a way, perhaps by a notation on the figure itself and/or re-wording the legend, to make this more clear. Finally, I was not clear what the 'categories' were that the fractions made reference to. The legend text was not clear to me. For instance, is it 5/7 that 'looked like that image'? If so, what did the others look like? Please clarify in the figure legend.

> We have attempted to clarify the legend and the labels within the figure, including by directly labeling the two columns of images as “Transgenic family 1/2". In essence there were two categories of interest: apices that shown distinct adaxial GUS signal and apices that did not. The reviewer is correct that 5 of 7 stained plants for transgenic family 1 for the 298 bp promoter construct matched the photograph shown, i.e. they had no detectable signal (Figure 5C, left-hand side). Faint adaxial-localized signal was visible for the other 2 stained plants (out of 7), as noted in the text.

4. Figure 7: The authors do a great job showing the true variability in this dataset. I can see that the means of shortest two promoters tend toward the ago7 mean, which the other promoters tend toward the mean of the wt line. But given the variability and relatively low numbers of replicates, are any of these trends statistically distinguishable? If not, is there value in this figure?

Despite the variability, mean leaf-blade length-to-width ratios for different genotypes are statistically distinguishable when all leaf positions are considered together. After accounting for the effect of leaf position with a linear term, lines transformed with transgenes including promoter fragments 422 bp or larger differ from the *ago7* mutant control line ($p < 0.001$, t-tests on linear contrasts) whereas lines with the 298 bp and 0 bp promoters did not ($p = 0.052$ and 0.851 respectively). These differences are consistent with partial complementation for transgenes including at least 422 bp of the *AGO7* promoter.

5. Figure 9: I felt that this figure extrapolated perhaps too far beyond the data shown. In particular, the present manuscript did not provide strong evidence that SPLs activate AGO7. It's possible because there are SPL-binding sites in the AGO7 promoter, but as the transgenics showed, their function in driving AGO7 expression is murky at best. It's a nice figure, and perhaps a useful hypothesis though .. perhaps consider adding a question mark next to the SPL -> AGO7 link?

We have added the suggested question mark to Figure 9 to indicate that it is uncertain whether SPLs do in fact transcriptionally activate AGO7.

6. Is there a succinct list of ALL the 'true hits' from the y1h experiments for all three genes? This could be most useful for people in the future who want to follow up. I strongly suggest this be added, perhaps a supplemental excel file, and briefly mentioned in the manuscript.

We have deposited a supplemental Excel file (Zenodo record 1472235) with ranked lists all TFs for each screen and added a brief note in the results section. The new file simplifies identification of TFs that fall above hit criteria such as the 6-median-absolute-deviations-above-plate-median cutoff emphasized in the paper.

7. Lines 257-259: Why describe and cite data/experiments that weren't used at all in the current study? I did not understand this. Consider removal.

We believe these data will be useful for developers of image analysis software and therefore wanted to make them discoverable and to make clear that they came from the experiment described in this paper. We have further shortened the description of the data in the Methods section, and we believe this brief mention is unobtrusive.

Reviewer #2:

Hoyer et al. have characterized the promoter of Arabidopsis AGO7 by combining a high throughput yeast-1-hybrid screening approach with in planta complementation assays. Their approach in yeast has identified members of the TCP and SPL transcription factors families as interacting with a specific fragment of the AGO7 promoter; they also identified cis-sequences that are necessary for the interaction with these transcription factors in yeast. When nested deletions of the promoter that harbour or not these sites were tested in planta for the expression pattern they produce and their ability to revert the ago7 mutant phenotype, it appeared that the identified TCP & SPL sites are dispensable and that functionally relevant segment(s) lay within a flanking region.

Although the results obtained do not fit a "simple model", the in vivo mapping of AGO7 promoter performed is significantly advancing our understanding of AGO7 transcriptional regulation. The manuscript is exemplary by the quality of its writing, the precision of the analyses, the interpretation of the results and the quality of the methods (eg. availability of all code used for analysis).

My only suggestion would be to clarify in 1 or 2 sentences why they did not pursue further the analysis of AGO1 and AGO10 promoters after the Y1H screen.

A note has been added to the results section. The *AGO7* promoter was prioritized simply because of interest in SPLs and TCPs and the ease with which candidate binding sites for those TFs could be predicted.
